# The clinical efficacy and safety of acupuncture intervention on cancer-related insomnia: A systematic review and meta-analysis

**DOI:** 10.3389/fnins.2022.1026759

**Published:** 2022-12-15

**Authors:** HaiXin Yu, CaiYun Liu, Bo Chen, JingBo Zhai, DongSheng Ba, Zheng Zhu, NingCen Li, PeiYong Loh, AoXiang Chen, Bin Wang, Yi Guo, YangYang Liu, ZeLin Chen

**Affiliations:** ^1^Department of Acu-moxibustion and Tuina, Tianjin University of Traditional Chinese Medicine, Tianjin, China; ^2^Acupuncture Research Center, Tianjin University of Traditional Chinese Medicine, Tianjin, China; ^3^Evidence-Based Medicine Center, Tianjin University of Traditional Chinese Medicine, Tianjin, China; ^4^National Clinical Research Center for Cancer, Tianjin Medical University Cancer Institute and Hospital, Tianjin, China

**Keywords:** acupuncture, cancer-related insomnia, systematic review, meta-analysis, PSQI, efficacy

## Abstract

**Objective:**

To evaluate the efficacy and safety of acupuncture in treating symptoms for Cancer-related Insomnia(CRI) patients.

**Methods:**

Seven databases were searched from the time of database establishment to 31 March 2022. Randomized Controlled Trials (RCTs) on acupuncture intervention for CRI were collected. Literature screening and data extraction were performed independently by two researchers. Meta-analysis was performed using RevMan 5.4 software.

**Results:**

A total of 13 articles with 1,109 participants were included. Five hundred and seventeen in the treatment group and 592 in the control group. Ten of the RCTs used the PSQI rating scale and four randomized controlled trials used the ISI rating scale, and the PSQI and ISI were analyzed together as continuous data. The results of the meta-analysis were: MD = −1.83, 95%CI = [−2.71, −0.94], *P* < 0.0001, indicating a significant improvement in PSQI scores in patients with CRI by acupuncture intervention; MD = 0.79, 95%CI = [−0.46, 2.03], *P* = 0.22. Acupuncture was not statistically significant on ISI scores for patients with CRI compared to controls, which does not yet indicate that acupuncture is effective for symptoms in patients with CRI. The results of the meta-analysis of the other 4 items using sleep disorder logs as efficacy analysis data were as follow, relative risk RR = 0.47, 95%CI = [0.33, 0.66], *P* < 0.0001. The difference was statistically significant, indicating that acupuncture can improve the symptoms of CRI patients compared to control group.

**Conclusion:**

Acupuncture can improve the symptoms of patients with CRI to some extent, but due to the relatively small number and low quality of the included literature in this study, more high-quality clinical trials are needed as supplement the evidences in future.

**Systematic review registration:**

https://www.crd.york.ac.uk/prospero/.

## Introduction

Cancer-related Insomnia (CRI) is also called tumor-related sleep disorder. It refers to the subjective experience of cancer patients who experience insufficient sleep time and sleep quality to meet normal physiological needs after the onset of cancer, thus affecting their daily life and health (Induru and Walsh, 2014; Zhuang and Fang, 2022). It is a more common clinical symptom, especially in patients with breast cancer, lung cancer and head and neck cancer (Induru and Walsh, 2014). Studies have shown that the incidence of CRI significantly exceeds that of the general insomnia population by a factor of two, accounting for 52.6–67.4% (Reilly et al., 2013), and the incidence of insomnia in cancer patients is 70.1% in China (Schieber et al., 2019). CRI is the second most urgent concomitant symptom of cancers after fatigue (Reilly et al., 2013). CRI affects the quality of life of most patients (Holtdirk et al., 2020) and prolonged insomnia can lead to greater physical and psychological damage, leading to many other serious problems, such as anxiety, depression and impairment of the body's immune function (Fleming et al., 2010; Yao and Tian, 2020), as well as other complications, such as obesity, hypertension, cardiovascular disease, etc. (Knutson et al., 2009). Most clinical studies have shown that most of the drugs are currently commonly used in clinical practice that approving by the Food and Drug Administration (FDA), such as benzodiazepines, anticonvulsants, antihistamines, and melatonin agonists (Asnis et al., 2015), and the treatment effect is often < 80% and many adverse effects (Asnis et al., 2015; Wilt et al., 2016; Lu and Guo, 2021), such as drug resistance, memory loss and dependence, etc. (Zhao, 2013). At the same time, not only does it take a long time to treat, but it also increases the financial burden on patients and their families, which affects the long-term survival of patients (Groenvold et al., 2007). Therefore, finding an effective and inexpensive alternative therapy has become an urgent task.

Acupuncture and moxibustion in traditional Chinese medicine has a long history, and its advantages of quit onset, simplicity and low side effects also have an irreplaceable role in modern treatment. As a non-pharmacological interventional technique, acupuncture has been shown to be beneficial for most patients from physical to psycho-spiritual aspects compared to other alternative therapies (Gould and MacPherson, 2001; Choi et al., 2017). Studies have shown that acupuncture can relieve pain, fatigue, hot flashes, anxiety and depression in cancer patients (Choi et al., 2012; Garcia et al., 2013; Posadzki et al., 2013). Therefore, acupuncture has gradually become one of the most popular treatment for patients (Gould and MacPherson, 2001). With the gradual increase of clinical studies using acupuncture for CRI in domestic and abroad, most of the meta-analysis were made for different therapies of acupuncture combined therapies of acupuncture and medicine or moxibustion (Chen, 2021; Yin et al., 2021; Wang et al., 2022; Zhuang and Fang, 2022). The results all showed that the combined therapy were more effective for CRI, but there were fewer studies on acupuncture alone, and no meta-analysis of acupuncture alone for CRI was found in recent years. Recently, some new randomized controlled trials were found to verify the efficacy of acupuncture for CRI (Lee et al., 2022) and meta-analysis was conducted for articles that met the study criteria to evaluate the efficacy and safety of acupuncture for CRI and to provide a medical reference for clinical treatment with acupuncture therapy.

## Methods and materials

### Retrieval policy

Using computer to search Chinese databases: Chinese National Knowledge Infrastructure (CNKI), WANFANG, VIP. English databases: PubMed, Web of science, Cochrane, Embase. Studies published from the establishment of the databases to 31 March 2022 were searched. The retrieval strategy of “subject words+free words” was adopted, The search terms used are as follows: [“acupuncture” or “electroacupuncture” or “transcutaneous electrical acupoint stimulation (TEAS) ”or “auricular acupuncture” or “needle warming moxibustion”] and [“Cancer-related Insomnia” or “tumor” or “cancer” or “neoplasia” or “CRI”] and [“sleep” or “insomnia” or “sleep disorder”]. The rest of the database is retrieved according to different retrieval methods. The search strategy and search process are detailed in the Supplementary material. PROSPERO registration has been completed in March 2022 with the registration number CRD42022309870.

### Inclusion criteria

(1) Research type: Randomized Controlled Trial (RCT). (2) Participants: all adults cancer patients met the diagnostic criteria of insomnia in *National Comprehensive Cancer Network* and *The Diagnostic and Statistical Manual of Mental Disorders*, 5th Edition (DSM-5), regardless of cancer type, stage or disease duration. (3) Intervention: acupuncture, electroacupuncture, transcutaneous electrical acupoint stimulation, auricular acupuncture, needle warming moxibustion, while western medicine, routine care or sham acupuncture were used in the control group. (4) Outcome index: the curative effect of acupuncture on CRI was measured by any validated tool. The curative effect evaluation index includes one of the following, PSQI, ISI, sleep efficiency, sleep disorder log and subjective self-report sleep questionnaires.

### Exclusion criteria

(1) Articles on the combination of acupuncture and medicine or the combination of acupuncture and moxibustion with other therapeutic interventions. (2) Insomnia was not caused by tumor or cancer. (3) The outcome indicators did not meet the inclusion criteria, the data and information are incomplete. (4) Repeated articles. (5) Articles with Jadad scores below 3 scores.

### Outcomes

Currently the most commonly outcome indicators are PSQI, ISI and sleep efficiency. PSQI can evaluate sleep quality, sleep dysfunction in clinical and non-clinical samples (Mollayeva et al., 2016). The secondary outcomes included ISI and effective rate. The ISI is used to assess the character and symptoms of the subject's sleep disorder. ISI has beneficial internal consistency, temporal stability and construct validity of instrument for diagnosing CRI patients (Savard et al., 2005). Effective rate included sleep disturbance after treatment, sleep information recorded with sleep diaries, or sleep quality assessed with other validated questionnaires. Security indicators include adverse events.

### Data extraction

Two researchers (CaiYun Liu and DongSheng Ba) independently screened articles that met the inclusion criteria and extracted data, and summarized the authors, publication year, subjects (gender-age characteristics, tumor type, sample size), intervention mode, control mode, outcome index and adverse events into an excel table. If the two researchers disagree, they will be reviewed by a third researcher (HaiXin Yu).

### Quality assessment of included studies

The evaluation of the literature was performed using the risk of bias assessment tool in the RevMan 5.4 software provided by the Cochrane Collaboration Network, which focuses on the following components: (1) Random sequence generation; (2) Allocation concealment scheme; (3) Blinding of participants and personnel; (4) Blinding of outcome assessment; (5) Incomplete outcome data; (6) Selective outcome reporting; (7) Other bias (Higgins et al., 2011). The above seven biases were assessed, with red representing high risk, yellow representing unclear risk, and green representing low risk.

### Statistical methods

Meta-analysis was performed using RevMan 5.4 software, and Relative Risk (RR) was used for efficacy analysis results; Mean Difference (MD) was used for continuous analysis results, and 95% confidence intervals (CI) were given. The *I*^2^ statistic (0–100%) was used to assess the heterogeneity between the results of studies expressing different intervention modalities, and when the statistical heterogeneity between studies was small (*P*>0.1, *I*^2^ < 50%), it indicated that the results were not statistically significant and a fixed-effects model was used. When there was a large statistical heterogeneity between studies (*P* < 0.1, *I*^2^ ≥ 50%), it indicated that the results were statistically significant, and the source of heterogeneity was identified and subgroup analysis was performed. When statistical heterogeneity existed between subgroups without significant clinical heterogeneity, a random-effects model was used and the results were analyzed.

## Results

### Study selection

**Figure 1:**
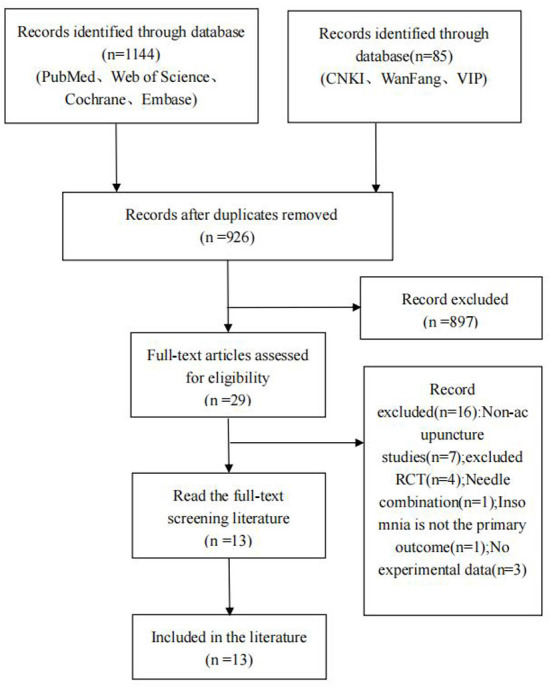
PRISMA flow diagram of included articles.

Through the retrieval of various databases, a total of 1,229 articles were identified from the preliminary searches, including 85 Chinese articles and 1,144 English articles. After eliminating duplicate articles (*n* = 303), remaining 926 articles, and after reading the titles and abstracts of the articles, the articles were excluded according to the requirements of the inclusion criteria (*n* = 897), 29 remaining articles were excluded after reading the full text (*n* = 16), and finally 13 RCTs were included, 3 in Chinese (Song et al., 2015; Peng et al., 2016; Shen et al., 2016) and 10 in English (Feng et al., 2011; Bokmand and Flyger, 2012; Frisk et al., 2012; Mao et al., 2014; Garland et al., 2017, 2019; Bao et al., 2021; Höxtermann et al., 2021; Zhang et al., 2021; Lee et al., 2022). The flow of trials outlined in Figure 1.

### Study information

The basic information in the articles were collected after reading the full text and plotted in Table 1, which included the author, year of publication, tumor type, gender-age characteristics, sample size, intervention, control modality, outcome indicators and adverse events. As known from the table, a total of 1,109 subjects were included in the 13 RCTs, including 517 in the treatment group and 592 in the control group. The treatment group included 3 treatment: electroacupuncture (Frisk et al., 2012; Mao et al., 2014; Shen et al., 2016; Garland et al., 2017; Bao et al., 2021; Zhang et al., 2021; Lee et al., 2022) (*n* = 7), manual acupuncture (Feng et al., 2011; Bokmand and Flyger, 2012; Song et al., 2015; Peng et al., 2016; Garland et al., 2019) (*n* = 5) and auricular acupuncture (Höxtermann et al., 2021) (*n* = 1). Of the 13 included articles, 11 (Feng et al., 2011; Mao et al., 2014; Song et al., 2015; Peng et al., 2016; Shen et al., 2016; Garland et al., 2017, 2019; Bao et al., 2021; Höxtermann et al., 2021; Zhang et al., 2021; Lee et al., 2022) provided results for PSQI and ISI as continuous data measures, of which 7 (Feng et al., 2011; Mao et al., 2014; Song et al., 2015; Peng et al., 2016; Shen et al., 2016; Garland et al., 2017; Höxtermann et al., 2021) had PSQI as the primary outcome indicator and 3 (Garland et al., 2019; Zhang et al., 2021; Lee et al., 2022) had ISI as the primary outcome indicator; 3 (Garland et al., 2019; Zhang et al., 2021; Lee et al., 2022) had PSQI as the secondary outcome indicator and 1 paper (Bao et al., 2021) had ISI as the secondary outcome indicator. Another four (Bokmand and Flyger, 2012; Frisk et al., 2012; Song et al., 2015; Peng et al., 2016) provided results for efficacy analysis measures.

**Table 1:** Basic information of the included literature.

**Reference**	**Age; Gender (T/C)**	**Cancer type**	**sample dose**	**Interventions**	**Control (regimen)**	**Outcomes**	**Adverse events**
Bao et al. (2021)	Age:60.3/62.7/57.3 Gender (F:M) : 20:7/ 19:5/ 21:3	13 types of cancer including breast cancer, colon cancer, lung cancer, ovarian cancer, and endometrial cancer	*n* = 75 T:27 C:24 UC:24	EA	Sham EA (Sham-Retractable nonpenetrating needles at non-acupoints); routine care	ISI	T: Adverse events were reported in 6 patients, 3 with pain at the acupuncture site, 2 with abrasions, and 1 with claustrophobia after wearing an eye patch
Bokmand and Flyger (2012)	Age:54.1/53. 4 Gender:F	breast cancer	*n* = 94 T:31 C:29 NT:34	Manual acupuncture	Sham acupuncture (superficial penetrating needles at non-acupoint); no treatment	Sleep disturbance (logged as yes or no)	T: 5 women had side effects, colds and sensitivities. C: 5 women had side effects including fatigue and joint tenderness.
Feng et al. (2011)	Age:63.8/63.6 Gender: (F:M) :14:26/13:27	7 types of malignant tumors including lung cancer, gastric cancer, lymphoma,breast cancer, colorectal cancer, and ovarian cancer	*n* = 80 T:40 C:40	Manual acupuncture	Fluoxetine capsules (20 mg/day for 30 days)	PSQI	None
Frisk et al. (2012)	Age:54.1/53.4 Gender:F	Breast cancer	*n* = 45 T:26 C:18	EA	Hormone therapy (tamoxifen/tolimifen)	Sleep Disorder Log (1) Hours of sleep per night; (2) Number of wake-ups during the night	None
Garland et al. (2017)	Age:52.9/50.4 Gender:F	Breast cancer	*n* = 58 T:30 C:28	EA	Gabapentin tablets (300 mg for 3 days,followed twice daily for 3 days, then thrice daily for rest 50 days (8 weeks totally)	PSQI,specific PSQI domain	None
Garland et al. (2019)	Age:62.3/60.7 Gender (F:M) :43:37/48:32	Breast cancer, prostate cancer, hematologic cancer	*n* = 160 T:80 C:80	Manual acupuncture	Cognitive behavioral therapy (5 weekly sessions, followed by 2 biweekly sessions,7 sessions totally over 8 weeks)	ISI, PSQI	T: Soreness, itching and pain at the acupuncture site (*n* = 9). C: Lethargy and daytime fatigue (*n* = 5)
Lee et al. (2022)	Age:57.63/62.33/61.38 Gender (F:M) :6:1:5/2:5:3	Breast, thyroid and other cancers	*n* = 22 T:8 C:6 UC:8	EA	Sham EA (a blunt end not penetrating the skin); routine care	ISI, PSQI	T: 2 cases of headache, 1 case of cough, low back pain, common cold, enteritis, dizziness, knee joint pain, rhinitis, and 1 case of dyspepsia. C: 2 cases of common cold,1 case of shoulder joint pain, skin allergy, lymphadenitis, hematuria, and 1 case of dyspepsia. UC: arthritis, skin spots, diarrhea, dyspepsia, toothache, and intestinal obstruction in 1 case each.
Mao et al. (2014)	Age:59.7 (41–76) Gender:F	breast cancer	*n* = 67 T:22 C:22 WLC:23	EA	Sham EA (nonpenetrating needles at non-acupoints); waiting list comparison	PSQI	T: tingling, numbness, pain at needling site (*n* = 6); C: 4 case.
Zhang et al. (2021)	Age:52.5/52.7 Gender:F	Breast cancer	*n* = 28 T:13 C:15	EA	waiting list comparison	ISI, PSQI	T: Soreness, itching and pain at the acupuncture site (*n* = 9), mild and moderate. C: Lethargy and daytime fatigue (*n* = 5),mild and moderate.
Peng et al. (2016)	Age:59.36/60.95 Gender: (F:M) :36/68	Tumor type unknown	*n* = 208 T:104 C:104	Manual acupuncture	Eszolam tablets (1mg/day for 7 days)	PSQI, Effective rate	None
Höxtermann et al. (2021)	Age:56.6/54.8 Gender:F	Breast cancer	*n* = 52 T:26 C:26	auricular acupuncture	Psychoeducation (1 session psychoeducation+insomnia advice booklet)	PSQI	T: 39 cases of bruising, pain, hot flashes, pressure sensitivity, etc.
Song et al. (2015)	Age:n.r Gender:n.r	Tumor type unknown	*n* = 120 T:60 C:60	Manual acupuncture	Eszolam tablets (1 mg/day for 7 days)	PSQI, Effective rate	None
Shen et al. (2016)	Age:54.9/58.1 Gender (F:M) : 28:22/31:19	Lung cancer	*n* = 100 T:50 C:50	EA	Analgesic drugs combined with zolpidem (10 mg/day for 4 weeks)	PSQI	None

N, total number of subjects, F, female; M, male; T, experimental group; C, control group; UC, usual care group; EA, electroacupuncture; NT, blank group; WLC, waiting list control group; n.r, not reported.

### Risk of bias and quality assessment

**Figure 2:**
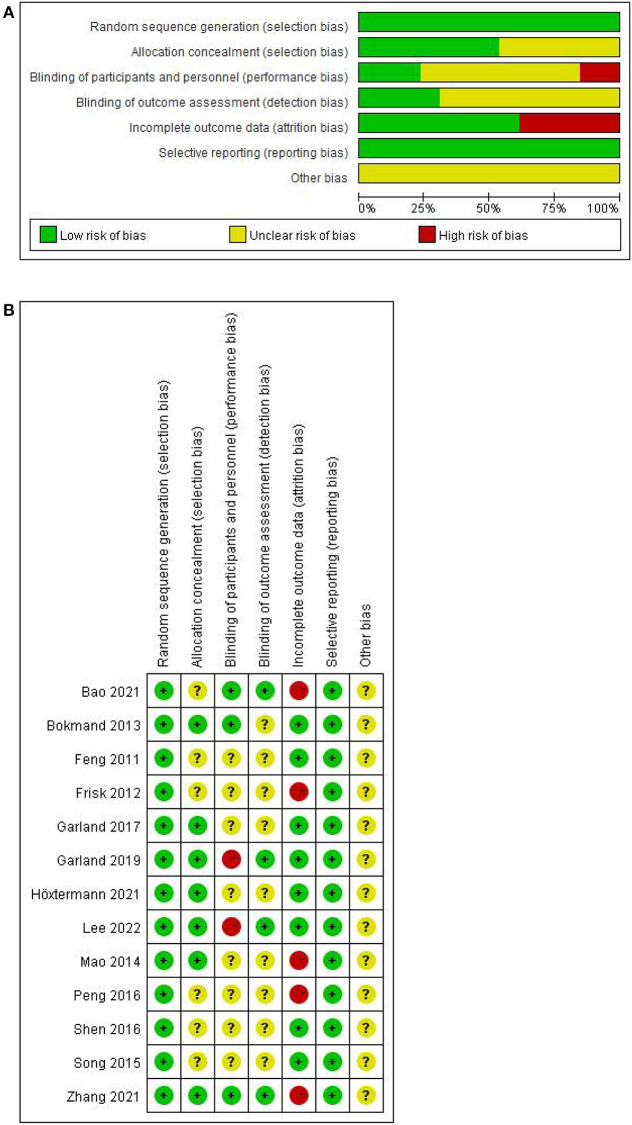
Risk of bias of included trials. **(A)** Assessment of risk of bias presented as percentages across all included studies. **(B)** Risk of bias summary for each included study.

All included RCTs correctly used the randomization allocation method, with 9 items (Feng et al., 2011; Bokmand and Flyger, 2012; Mao et al., 2014; Song et al., 2015; Peng et al., 2016; Shen et al., 2016; Garland et al., 2017; Höxtermann et al., 2021; Zhang et al., 2021) using random number tables, 2 items (Frisk et al., 2012; Lee et al., 2022) using stratified randomization, and 2 items (Garland et al., 2019; Bao et al., 2021) using random squares. Seven items (Bokmand and Flyger, 2012; Mao et al., 2014; Garland et al., 2017, 2019; Höxtermann et al., 2021; Zhang et al., 2021; Lee et al., 2022) mentioned allocation concealment (six Bokmand and Flyger, 2012; Mao et al., 2014; Garland et al., 2017, 2019; Zhang et al., 2021; Lee et al., 2022 for opaque or closed envelope hiding and one Höxtermann et al., 2021 for central random hiding), and the rest were not mentioned. Three items (Bokmand and Flyger, 2012; Bao et al., 2021; Zhang et al., 2021) were blinded to subjects and researchers. Four items (Garland et al., 2019; Bao et al., 2021; Zhang et al., 2021; Lee et al., 2022) implemented blinding of outcome assessors. Five items (Frisk et al., 2012; Mao et al., 2014; Peng et al., 2016; Bao et al., 2021; Zhang et al., 2021) reported cases missing visits or active withdrawals, of which the number and reasons for missing visits or withdrawals were unbalanced, and the remaining eight (Feng et al., 2011; Bokmand and Flyger, 2012; Song et al., 2015; Shen et al., 2016; Garland et al., 2017, 2019; Höxtermann et al., 2021; Lee et al., 2022) had no personnel withdrawal. Selective reporting bias was low. For the presence of other biases, none of the 13 RCTs mentioned (Figure 2).

### Primary outcome

**Figure 3:**
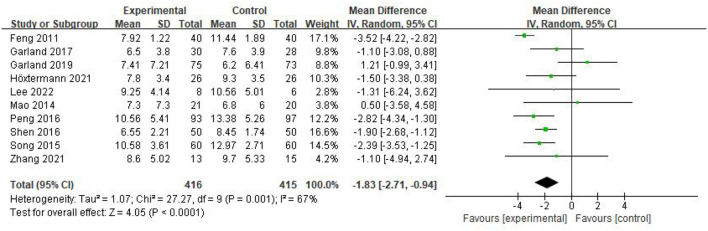
Forest plot of PSQI scale scores.

PSQI: Ten of the included studies (Feng et al., 2011; Mao et al., 2014; Song et al., 2015; Peng et al., 2016; Shen et al., 2016; Garland et al., 2017, 2019; Höxtermann et al., 2021; Zhang et al., 2021; Lee et al., 2022) addressed changes in PSQI before and after treatment, with a total of 831 participants, of whom 416 were in the experimental group and 415 in the control group. The heterogeneity test was first performed with *P* = 0.001 and *I*^2^ = 67%, showing a large heterogeneity between studies, and a random effects model was adopted for meta-analysis. The results showed that the effect size MD = −1.83, 95% CI = [−2.71, −0.94], *P* < 0.0001. The difference was statistically significant, indicating acupuncture was effective in treating patients with CRI compared to the control group (Figure 3).

**Figure 4:**
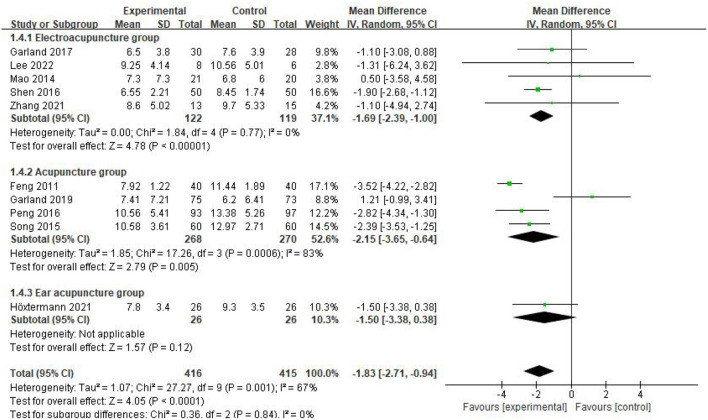
Subgroup analysis of PSQI scores.

Subgroup analysis was performed according to the different intervention methods, and the results of the subgroup analysis showed that the heterogeneity among the three subgroups was *P* = 0.84, *I*^2^ = 0%, indicating that there was no heterogeneity among the subgroups, and the effect size after the three subgroups were combined was *P* = 0.001, *I*^2^ = 67%, indicating that there was heterogeneity. As we can see from the figure, the literature data on manual acupuncture for patients with CRI may be a source of heterogeneity, so a random effects model was used for analysis, MD = −1.83, 95%CI = [−2.71, −0.94], *P* < 0.0001. The difference is statistically significant, indicating that acupuncture treated CRI patients with better symptoms than the control group (Figure 4).

### Secondary outcome

**Figure 5:**
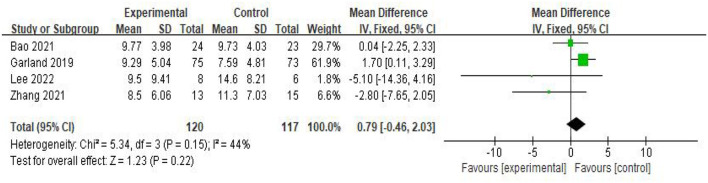
Forest plot of ISI scale scores.

ISI: Four of the included studies (Garland et al., 2019; Bao et al., 2021; Zhang et al., 2021; Lee et al., 2022) addressed the changes in ISI before and after treatment in a total of 237 participants, of which 120 were in the experimental group and 117 in the control group. A heterogeneity test was first performed with *P* = 0.15, *I*^2^ = 44%, so a fixed-effects model was adopted for meta-analysis. The results showed that the effect size MD = 0.79, 95%CI = [−0.46, 2.03], *P* = 0.22. The ISI scores of patients with CRI in the treatment group intervention were not statistically significant compared to the control group (Figure 5).

**Figure 6:**
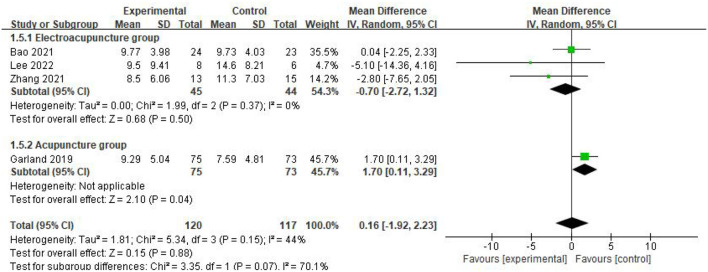
Subgroup analysis of ISI scores.

Subgroup analysis was performed according to the different intervention methods and the results of the subgroup analysis showed that the heterogeneity between the two subgroups was *P* = 0.15, MD = 0.16, *I*^2^ = 44%, indicating that there was no heterogeneity between the two subgroups, and the effect size after the combination of the two subgroups was *P* = 0.07, *I*^2^ = 70.1%, indicating that there was heterogeneity. As can be seen from the figure, literature data on electroacupuncture for CRI patients may be a source of heterogeneity, so meta-analysis was performed using a random effects model, MD = 0.16, 95%CI = [−1.92, 2.23], *P* = 0.88 indicating that acupuncture improved ISI decline in CRI patients compared to controls without statistical significance (Figure 6).

### Efficacy analysis

**Figure 7:**
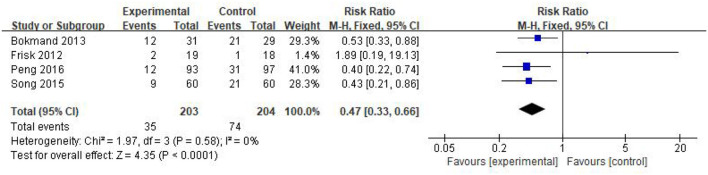
Forest plot of dichotomous data.

Four of the included studies (Bokmand and Flyger, 2012; Frisk et al., 2012; Song et al., 2015; Peng et al., 2016) reported changes before and after sleep disorders in a total of 407 participants, including 203 in the experimental group and 204 in the control group. A heterogeneity test was first performed with *P* = 0.58, *I*^2^ = 0%, and a fixed-effect model was adopted for meta-analysis, which showed that acupuncture improved CRI patients better than controls in terms of sleep disturbances (RR = 0.47, 95%CI = [0.33, 0.66], *P* < 0.0001) (Figure 7).

### Summary of acupuncture treatment and security analysis

The acupuncture treatment sessions varied among studies (Table 2). The most commonly used acupoints were: Baihui (GV20), Shenting (GV24), Neiguan (PC6), Sishencong ((EX-HN1), Yintang (EX-HN3), Zusanli (ST36), all of which were used at least three times, with a possible selection of 2-12 points in each study, and a minimum treatment duration of 1 week and a maximum of 10 weeks, ranging from 20 to 30 minutes. Another study chose auricular acupuncture (Höxtermann et al., 2021) with a duration of 33 days, shorter than manual acupuncture and electroacupuncture. The most common adverse effects of acupuncture in the included studies were pain, pruritus, bruising, and colds. Comparison the relatively low number of adverse events in the experimental group compared to the control group indicates that acupuncture has fewer side effects in the treatment of CRI and has advantages in terms of safety compared to other treatments (e.g., sham acupuncture and cognitive behavioral therapy).

**Table 2:** Summary of acupuncture treatment.

**Include literatures**	**Acupuncture type**	**Acupoints**	**Treatment regimen**
Bao et al. (2021)	EA	Hegu (LI4), Neiguan (PC6), Houxi (SI3), Taichong (LR3), Xiaxi (GB43), Fenglong (ST40), Bafeng 2, Bafeng 3	Biweekly for first 2 weeks, then weekly for 6 weeks;30 min/session;10 sessions totally
Bokmand and Flyger (2012)	Manual acupuncture	Neiguan (PC6), Sanyinjiao (SP6), Taichong (LR3), Taixi (KI3)	Once weekly for 5 wks;15–20 min/session;5 sessions totally
Feng et al. (2011)	Manual acupuncture	Sishencong (EX-HN1), Baihui (GV20), Yintang (EX-HN3), Neiguan (PC6), Shenmen (HT7), Fenglong (ST40), Sanyinjiao (SP6), Yinlingquan (SP9), Xuehai (SP10)	Once daily for 30 days;20–30 min/session;30 sessions totally
Frisk et al. (2012)	EA	Baihui (GV20), Xinshu (BL15), Shenshu (BL23), Ciliao (BL32), Neiguan (PC6), Shenmen (HT7), Yinlingquan (SP9), Sanyinjiao (SP6), Taichong (LR3)	Twice weekly for 2 weeks, followed by once weekly for 10 weeks;30 min/session;14 sessions totally
Garland et al. (2017)	EA	Taixi (KI3), Sanyinjiao (SP6), plus Guanyuan (CV4) if supine position or Shenshu (BL23) if prone position;and up to 4 supplemental points	Twice weekly for 2 weeks, followed by once weekly for 6 weeks;30 min/ session; 10 sessions totally
Garland et al. (2019)	Manual acupuncture	Shenmen (HT7), Sanyinjiao (SP6)	Twice weekly for 2 weeks, followed by once weekly for 6 weeks;30 min/ session;10 sessions totally
Lee et al. (2022)	EA	Baihui (GV20), Yintang (EX-HN3), Neiguan (PC6), Shenmen (HT7), Jinmen (BL63), Dazhong (KI4), and 4 more additional points	2–3 times a week for 4 weeks, for 30min/session, 10 sessions totally
Mao et al. (2014)	EA	distant acupoints plus at lease 4 local acupoints around the most painful joint	Twice weekly for 2 weeks, followed by once weekly for 6 weeks;30 min/ session;10 sessions totally
Zhang et al. (2021)	EA	Shenting (GV24), Baihui (GV20), Sishencong (EX-HN1), Neiguan (PC6), Sanyinjiao (SP6)), Taixi (KI3), and 4 additional points	Twice weekly for 6 weeks;25 min/session;12 sessions totally
Peng et al. (2016)	Manual acupuncture	Baihui (GV20), Shenting (GV24), Yintang (EX- HN3), Shenmen (HT7), Zusanli (ST36), Sanyinjiao (SP6)	Once daily for1 week; 30 min/session
Höxtermann et al. (2021)	Auricular acupuncture	Postantitragal belt, helix channel, shen men. Additional points were used to address comorbid symptoms	Twice weekly for 5 weeks; 20 min/session; 10 sessions totally
Song et al. (2015)	Manual acupuncture	Shenting (GV24), Baihui (GV20), Yintang (EX-HN3), Shenmen (HT7), Zusanli (ST36), Sanyinjiao (SP6)	Once daily for 7 days;30 min/session;7 sessions totally
Shen et al. (2016)	EA	Yintang (EX-HN3), Sishencong (EX-HN1), Anmian (EX- HN16), Qihai (CV6), Hegu (LI4), Quchi (LI11), Shenmen (HT7), Zusanli (ST36), Zhaohai (KI6), Shenmai (BL62), Taichong (LR3), and additional points	Once daily for 4 weeks;30 min/session;28 sessions totally

EA, electroacupuncture.

## Discussion

Cancer-related insomnia should belong to the category of “insomnia” from the perspective of Traditional Chinese Medicine, and “insomnia” can be traced back to the “*The Yellow Emperor's Canon of Internal Medicine”*. CRI is due to the fact that after chemoradiotherapy drugs enter the human body, the struggle between anti-pathogenic *qi* and pathogenic factors, resulting in disharmony between *yin* and *yang*, which leads to insomnia. In addition, the tumor itself belongs to the deficiency of the essence and excess, mostly due to the intertwined phlegm and blood stasis, which affects the function of viscera, the phlegm mists the heart, and the blood stasis stagnates the *qi* movement (Lu and Guo, 2021). Acupuncture therapy has the effect of regulate and harmonize *yin* and *yang*, dredging the meridians, and exerting its effect through related acupoints. Modern research has shown that the pathogenesis of insomnia is complex and closely related to the central nervous system (Bonnet and Arand, 1997), and studies have shown that patients with insomnia often over-active the sympathetic nerves during sleep thus accelerating metabolism in the body (Liu et al., 2022), what's more, acupuncture can affect sleep by activating parasympathetic nerves and inhibiting sympathetic nerves (Li et al., 2003; Liu et al., 2022). In addition, acupuncture can regulate central neurotransmitters, immune cytokines (Li, 2021), a series of chemical factors (Wei et al., 2021) and antioxidant defense systems (Li, 2021) to promote the restoration of balance between *yin* and *yang* in the body and achieve improved sleep.

Meta-analysis of 11 RCTs studies of acupuncture for the treatment of symptoms in patients with CRI showed that acupuncture was effective in improving sleep disturbance and reducing PSQI scores, but it could not be stated whether it could reduce ISI scores, due to the small sample size included in this study and the low quality of the articles. In addition, the type of tumor included in the study was not homogeneous, which could have an impact on the results. The control group of RCTs selected for the studies had not only sham acupuncture, but also a variety of interventions such as western medicine and routine care, which may have caused some error in the results. In terms of outcome indicators, the use of sleep logs and questionnaires as evaluation indicators in some RCTs may also have some limitations on the final results. In addition, in terms of adverse events, seven of the included articles (Bokmand and Flyger, 2012; Mao et al., 2014; Garland et al., 2019; Bao et al., 2021; Höxtermann et al., 2021; Zhang et al., 2021; Lee et al., 2022) mentioned mild to moderate reactions with different symptoms, such as acupuncture pain, cough, skin allergy, bruising and so on. In the subgroup analysis results showed that acupuncture or electroacupuncture had more positive efficacy and higher safety compared to drugs such as eszopiclone and gabapentin for the treatment of CRI, while some articles were less good and there were no good solutions mentioned in the text for these adverse reactions, which is something we need to improve in the future when using acupuncture therapy. The study shows that the probability of selecting these acupoints Baihui, Shenting, Neiguan, Sishencong, Yintang, and Zusanli is high, which may indicate that these acupoints have some improvement effect on the treatment of CRI, but the specific acupuncture protocol is not clear, and further investigation on the selection of acupuncture points is needed in the future. Moreover, although acupuncture treatment has fewer side effects, there is no clear solution to these adverse effects, which is what we need to improve in the use of acupuncture in the future, such as pain, itching, bruising, colds, etc. We found the funnel plot was highly biased (Supplementary Figure S1), we think the main reasons are as follows: Firstly, interventions are complex; Secondly, different acupoints selection. Therefore, reducing bias greatly is also an area for us to improve in the future.

Limitations of the study: (1) The reason of large heterogeneity in meta-analysis may be due to the diversity of intervention methods, as well as differences in the types of drugs, doses, acupuncture times, and acupuncture point selection in the control group. (2) The different types of cancer in studies, included types of breast cancer, thyroid cancer, colon cancer, and lung cancer, may also lead to different analysis results. In order to obtain better efficacy of acupuncture for CRI, future clinical trials can be conducted on different acupuncture interventions, compatibility of acupuncture point selection, needle retention time, and different cancer types and periods of onset to determine the clinical benefits of acupuncture for this disease.

## Conclusion

This study shows that acupuncture can improve the symptoms of CRI patients to some extent, sleep quality evaluated by PSQI improved, which is clinically useful and safe, and provides a basis for clinical treatment. Empirically, if combined with acupuncture and pharmacotherapy or alternative therapies, there may be a greater improvement in improving sleep quality. In terms of the safety of acupuncture efficacy, there is a need to further improve and ameliorate the adverse effects associated with acupuncture. However, because of the small amount and low quality of data included in this study, more large samples and high-quality clinical trials are needed to supplement the literature.

## Data availability statement

The datasets presented in this study can be found in online repositories. The names of the repository/repositories and accession number(s) can be found in the article/Supplementary material.

## Author contributions

Thesis guidance: BC, JZ, AC, BW, YG, YL, and ZC. Essay writing: HY, CL, and ZZ. Data analysis and collation: HY, CL, and DB. Illustration of the paper: HY and NL. Table design: HY and ZZ. Thesis translation: PL and HY. All authors contributed to the article and approved the submitted version.
